# A systematic review of published literature on mosquito control action thresholds across the world

**DOI:** 10.1371/journal.pntd.0011173

**Published:** 2023-03-03

**Authors:** Vindhya S. Aryaprema, Madeline R. Steck, Steven T. Peper, Rui-de Xue, Whitney A. Qualls

**Affiliations:** Anastasia Mosquito Control District, St. Augustine, Florida, United States of America; University of Heidelberg, GERMANY

## Abstract

**Background:**

Despite the use of numerous methods of control measures, mosquito populations and mosquito-borne diseases are still increasing globally. Evidence-based action thresholds to initiate or intensify control activities have been identified as essential in reducing mosquito populations to required levels at the correct/optimal time. This systematic review was conducted to identify different mosquito control action thresholds existing across the world and associated surveillance and implementation characteristics.

**Methodology/Principal findings:**

Searches for literature published from 2010 up to 2021 were performed using two search engines, Google Scholar and PubMed Central, according to PRISMA guidelines. A set of inclusion/exclusion criteria were identified and of the 1,485 initial selections, only 87 were included in the final review. Thirty inclusions reported originally generated thresholds. Thirteen inclusions were with statistical models that seemed intended to be continuously utilized to test the exceedance of thresholds in a specific region. There was another set of 44 inclusions that solely mentioned previously generated thresholds. The inclusions with “epidemiological thresholds” outnumbered those with “entomological thresholds”. Most of the inclusions came from Asia and those thresholds were targeted toward *Aedes* and dengue control. Overall, mosquito counts (adult and larval) and climatic variables (temperature and rainfall) were the most used parameters in thresholds. The associated surveillance and implementation characteristics of the identified thresholds are discussed here.

**Conclusions/Significance:**

The review identified 87 publications with different mosquito control thresholds developed across the world and published during the last decade. Associated surveillance and implementation characteristics will help organize surveillance systems targeting the development and implementation of action thresholds, as well as direct awareness towards already existing thresholds for those with programs lacking available resources for comprehensive surveillance systems. The findings of the review highlight data gaps and areas of focus to fill in the action threshold compartment of the IVM toolbox.

## Author summary

Many different methods are used and new methods are evolving to control nuisance and vector mosquito populations across the world. Implementation of such control methods at the optimal time has been identified as critical to maintaining the populations below unacceptable levels. The establishment of evidenced-based action thresholds is thus encouraged to achieve maximum results under available resources. This review was conducted to identify different action thresholds published worldwide since 2010 and to understand the associated surveillance characteristics. Only 87 publications filtered through the initial database identification of 1,485 publications were included in the review. Thirty inclusions reported originally generated thresholds, 13 inclusions reported a statistical method with a computable action threshold model, and 44 inclusions mentioned only previously generated thresholds that were used in their study. Overall, the inclusions with thresholds that directly related to disease transmission conditions (“epidemiological thresholds”) outnumbered the inclusions with thresholds that directly related to mosquito abundance (“entomological thresholds”). The majority of the thresholds originated in Asia targeting dengue and dengue vectors. The review discusses the associated surveillance characteristics such as the possibility of using external data (previously recorded data), spatial and temporal variations in data used, and the importance of an adequate lead time to initiate control interventions that would help guide to plan better surveillance programs for the development of action thresholds.

## Introduction

There are about 3,500 identified mosquito species in different geographic/climatic regions in the world. Some species act as human disease vectors while other species cause only biting nuisance problems. Vector-borne diseases account for more than 17% of all infectious diseases, causing more than 700,000 deaths annually [1]. More than 80% of the global population is at risk of vector-borne diseases, with mosquito-borne diseases (MBDs) being the largest contributor [2]. MBDs such as dengue, Zika, chikungunya, West Nile virus (WNV), eastern equine encephalitis (EEE), western equine encephalitis (WEE), and malaria cause major public health issues across the world. The global burden of malaria has been estimated to be 219 million cases, with more than 400,000 deaths every year [1], and reported 627,000 deaths in 2020 alone [3]. More than 3.9 billion people in over 129 countries are at risk of contracting dengue, with an estimated 96 million symptomatic cases and an estimated 40,000 deaths every year [1]. WNV is the most common MBD in the United States [4]. A total of 21,869 confirmed or probable cases of WNV disease were reported across all 50 states during 2009–2018 [5]. Chikungunya and Zika have caused the average yearly loss of over 106,000 and 44,000 disability-adjusted life years (DALYs) worldwide, respectively, between 2010 and 2019 [6]. There is a significant economic burden of MBDs as well. The direct global costs of malaria have been estimated to be at least US $12 billion per year [7]. The estimated total global cost of dengue was US $8.9 billion in 2013 [8]. The average cost of WNV in the United States was equivalent to an approximate average of $56 million per year during 1999–2012 [9,10]. There is no specific drug or vaccine available for MBDs such as dengue, chikungunya, etc. [11,12]. Control of vector populations is often the best way, if not the only way, to control the transmission of these diseases. On the other hand, although the major transmission of diseases such as malaria and lymphatic filariasis is controlled by mass drug administration (MDA), the vectors of the diseases are still a human nuisance. For all those reasons, mosquito control is still an integral constituent of the public health infrastructure of many countries.

There are many illustrious instances in history that mosquito control has effectively reduced disease burdens; reduction of malaria in the 1950s-1960s in some countries (in the Americas, Europe and Asia), dengue and yellow fever in the 1950s-1960s in the Americas, dengue in the 1970s-1980s in Singapore and dengue in the1980s-1990s in Cuba [13]. However, rapid and aggressive changes in mosquito population dynamics imposed by changing influential factors such as the environment, climate, geography, demographics and social culture, have been making mosquito control more challenging in the modern-globalized world. Development of resistance against all major classes of insecticides, and a shortage of effective insecticide innovation, registration, and supply have become another threat to mosquito control [14]. Compounding all those complexities, mosquito control is often reactive [14] as it is implemented only in direct response to already increased risk based on vector or disease surveillance results in the present time [15]. The effects of such control programs would be too late to prevent undesirable levels of human-mosquito contact, and thus the potential for disease explosions. One of the key elements lacking in many mosquito control programs is the use of reliable preemptive indicators to initiate proactive control operations before mosquito populations or disease risks are elevated to undesirable levels. It is widely accepted that Integrated Vector Management (IVM) and Integrated Mosquito Management (IMM) programs that make reliable evidence-based control decisions and that utilize appropriate control tools, can effectively reduce mosquito abundance and disease risk [16]. Therefore, early warning systems with evidence-based action thresholds that trigger interventions at the optimal time are now becoming critical in operational mosquito control. In addition to being more effective in controlling mosquito populations, such systems would help ensure prioritized allocation of resources, manage insecticide resistance, and hence help manage overall program logistics.

Understanding the relationship between influential factors and the dynamics of mosquito populations and disease transmission through properly planned surveillance systems is fundamental in establishing evidence-based mosquito control action thresholds. The structure of surveillance systems and therefore the data collected by different mosquito control programs are widely varied due to differences in geography, climate, economy, and other logistic factors. Therefore, there could be different action thresholds for the same species or species groups in different geographic/climatic regions as well as in the same region. Given the critical importance of the use of action thresholds in mosquito control, we systematically reviewed the published literature to identify different mosquito control action thresholds existing across the world and associated surveillance and implementation characteristics. The findings will give insights to mosquito control program managers and others, who anticipate developing and establishing their own action thresholds, to plan appropriate surveillance programs. Otherwise, this review will be helpful for those that wish to utilize already developed action thresholds due to the inability to establish comprehensive surveillance systems, especially the routine collection of mosquito data.

## Methods

**Fig 1:**
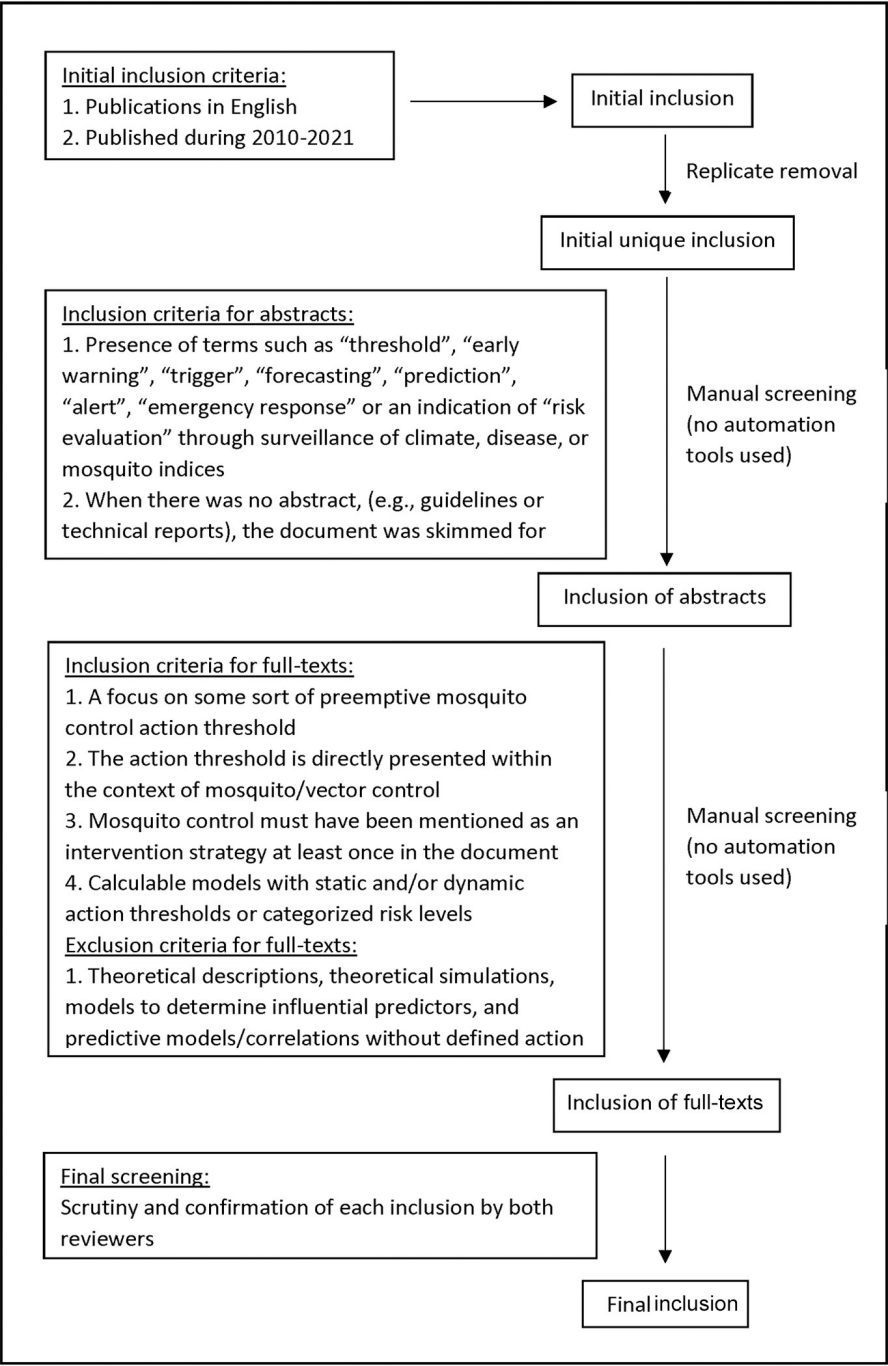
Search process and inclusion/exclusion criteria.

The systematic review was conducted following the PRISMA 2020 guidelines [17]. A protocol was prepared and agreed upon by all authors. An appropriate literature search was conducted from March 2020 to July 2021 using two main public search engines, Google Scholar (GS) and PubMed Central (PMC). The search terms were chosen to encompass a broad range of major vector species, diseases, surveillance/threshold measures, and analogies for “threshold”. Primary search queries involved various combinations of key terms using (i) major vector mosquito genera (“*Anopheles*”, “*Aedes*”, “*Culex*”), followed by AND “mosquito” AND “[] threshold” ([]: risk, epidemic, disease, tolerance, action), (ii) [(disease name) control” (disease name: malaria, dengue, zika, chikungunya, West Nile virus) followed by AND “threshold” /”action threshold”. (iii) “mosquito control”, “mosquito abundance”, “evidence based” with “threshold”/“action threshold”/”tolerance threshold”, and (iv) “control” followed by AND “mosquito” AND some analogous “threshold” terms that were discovered at the initial planning and search stage such as “trigger”/“warning”. For GS searches, all key terms were put in quotation marks to ensure a feasible number of search results (i.e. no more than several hundred). Quotation marks were also used for PMC searches; however, several PMC searches were conducted without quotation marks to instead broaden the search net after receiving any output of “no results found”. Searches were independently conducted by two human reviewers (VSA, MRS) with separate search terms until all planned combinations were exhausted and no new links appeared on the first listed page of results, considered as our point of saturation. Search process and the inclusion/exclusion criteria are summarized in Fig 1. Any disagreement at each stage was resolved by in-person discussions.

Direct control thresholds/operational targets to keep mosquito populations below unaccepted levels of biting nuisance or disease transmission, risk level indicators to guide mosquito control, defined pest levels, and indicators of seasonal mosquito activity were identified as mosquito control action thresholds in this review. Retrieved publications were divided into three inclusion categories to reflect the various circumstances an action threshold was implicated in them; (i) “generated thresholds”- publications with an originally generated action threshold, (ii) “generated-models”—publications with a statistical model that involved a computable trigger/risk assessment with input of dynamic data to indicate if the area under study is over a threshold or within a risk level, and (iii) “mentioned thresholds”–publications with clear mention of a previously generated action threshold/s when the cross-references cited in them were not eligible to be included in (i) or (ii). Within those three categories, two types of action thresholds were identified: “entomological thresholds”- the threshold directly indicates mosquito abundance without connecting it to an epidemiological indication (EI) of a disease (could be associated with either vectors or non-vector nuisance mosquitoes), and “epidemiological thresholds”- the threshold is directly related to an EI (transmission, outbreak, epidemic). Calculation methods of EIs greatly differed between publications, some calling for flexibility dependent on the choices of local government bodies, others using specific calculations. Definitions for EI that were defined and/or justified in publications are included alongside action threshold descriptions in supplemental data tables (S1, S2 and S3 Tables). Other data points extracted during the full-text screening included publication type (scientific journal, guidelines, theses/dissertations, etc., geographic origin of the threshold/s (continent, climatic region), spatial unit of analysis (study area size), spatial unit of implementation (suggested/planned area associated with threshold), origin of data (self-sampled data, previously recorded data), time span of used data, data frequency (temporal resolution used data), mosquito species in concern, disease in concern, variables used in the threshold, types of mosquito data (e.g. larval indices, adult trap counts), data frequency, associated proactive lead time from the exceedance of the threshold to its indication, self-assessment of the validity of the threshold, and intended use of the threshold.

## Results

**Fig 2:**
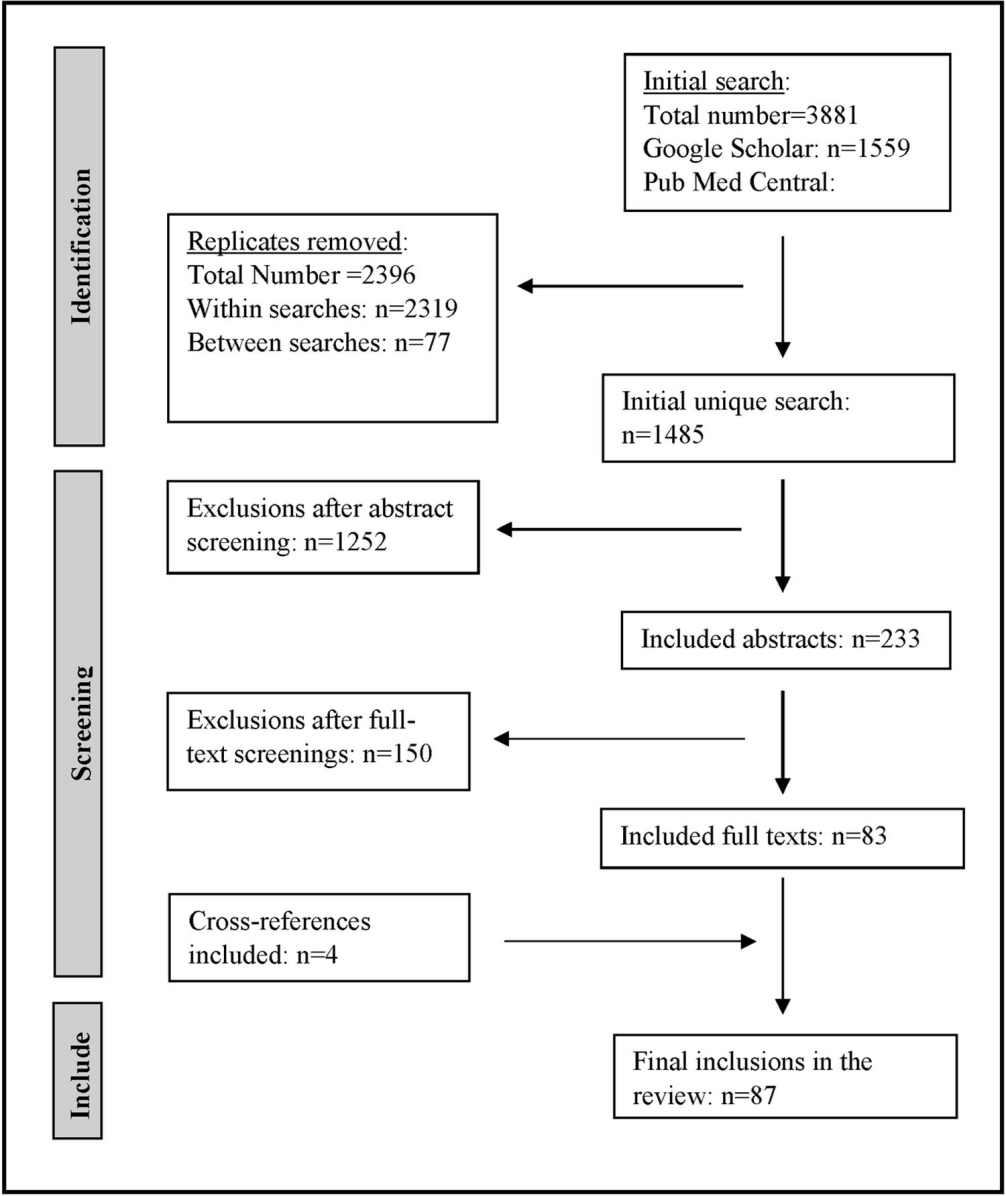
Flow diagram for included publications of the two search engines (Google Scholar and PubMed Central) to identify mosquito control action thresholds reported across the world.

GS and PMC produced 1,559 and 2,322 search results, respectively, between the two human reviewers and search query combinations. The quantitative value of unique search results was revealed by the removal of replicates through the compilation of all citations from recorded search queries. Replicate overlap between the two search engines was minimal and the total number of initial unique search manually screened for abstracts was 1,485. The abstract screening reduced the initial unique 1,485 publications to 233 publications for the next screening (i.e., reading the full text) and data extraction process. Combined with 4 cross-references found from publications that mentioned previous thresholds and that met all the inclusion criteria, the final screening identified 87 publications to be included in the review (inclusions, hereafter) (Fig 2).

There were 30 inclusions with “generated thresholds” (hereafter GTs, S1 Table) [18–47], 13 inclusions with “generated-models” (hereafter GMs, S2 Table) [48–60], and 44 inclusions with “mentioned thresholds” (hereafter MTs, S3 Table) [61–104]. Some of the MTs were evidence-based generations of previously (before 2010) published studies while a few did not discuss how they were generated. As per the respective inclusions, they were still in use and therefore, worthwhile looking into available relevant data points. Outcomes of extracted data points were presented as percentages of all inclusions or GTs/GMs and MTs as appropriate.

### 1. Publication type

The majority of inclusions were retrieved from scientific journals (89.7% [78/87]) [18–47 (GT); [48–59] (GM); 61–96 (MT)] and there were three program guidelines (3.4% [3/87]) [97–99 (MT)], three technical reports (3.4% [3/87]) [100–102 (MT)], two conference proceedings (2.3% [2/87]) [103,104 (MT)], and one thesis/dissertation (1.1% (1/87)] [60 (GM)].

### 2. Geographic origin of the threshold

Inclusions with thresholds were identified in broadly defined two climatic regions: tropical/sub-tropical (71.3% [62/87]) [18–44 (GT);49,51–60 (GM); 61,64,65,67,69,71,75,76,77,80,82,83,85,87–90, 92–98 (MT)], and temperate (24% [21/87]) [45–47 (GT); 48,50 (GM); 62,63,66,68,70,72–74,78,86,99–104 (MT)]. There were a few inclusions (5.7% [5/87]) that reported (i) a threshold developed from data of different climatic regions [48 (GM)], (ii) different thresholds from different regions [81 (MT)], and (iii) those that did not specify the geographic origin of the threshold [79,84,91 (MT)].

The highest number of inclusions were from Asia (36.7% [32/87]) [22–27,29,30,33–38,41–44 (GT); 56–59 (GM); 71,75,77,82,85,87,89,90,93,98 (MT)], followed by North America (27.5% [24/87]) [28,45,46 (GT); 48–51 (GM); 61–63,68,70,72,74,78,80,83,86,92,96,97,99,103,104 (MT)], and South America (14% [12/87]) [19,40 (GT); 52–55 (GM); 64,65,67,69,75,81 (MT)].

### 3. Threshold types and epidemiological indication (EI)

The majority of inclusions reported “epidemiological thresholds” (77% [67/87]) [20–44 (GT); 49–60 (GM); 61–65,67,69,71,73,75,76,77,79,81–84,87–91,93–95,97,98,101–104 (MT)], while only 19 inclusions reported “entomological thresholds” (21.8% [19/87]) [18,19,45–47 (GT); 48 (GM); 66,68,70,72,74,78,80,85,83,86,96,99,100 (MT)]. There was one inclusion that reported both types (1.1% [1/87]) [92 (MT)].

Disease transmission was the expected EI of most of the “epidemiological threshold” inclusions (56% [38/68]) [27–35,38,39,42–44 (GT); 50,55,58,59 (GM); 62,63,65,67,69,73,75,76,79,81,87,89,90–95,98,101 (MT)] followed by epidemics (28% [19/68]) [20,21,24,41 (GT); 49,52–54,57 (GM); 48,71,77,82,84,88,97,102–104 (MT)] and outbreaks (14.7% [10/68]) [22,23,26,36,39,40 (GT); 51,56,60 (GM); 64 (MT)]. There was one inclusion that reported thresholds for both disease transmission and outbreaks [25 (GT)]. However, it is noteworthy that the definition of the EI of those thresholds could be greatly varied between different inclusions.

### 4. Targeted mosquito species and diseases

*Aedes aegypti* Linn. and/or *Aedes albopictus* Skuse were the most reported mosquito species (80.5% [71/87]) [18,19,21–28,30,31,33–36,38–44,47 (GT); 49–60 GM), 61–64,66–71,73,75–95,98,99,102 (MT)]. Twelve inclusions mentioned *Culex* species (13.8% [12/87]) [20,29,45,46 (GT); 48 (GM); 63,72,74,97,100,103,104 (MT)] and two inclusions reported thresholds for *Anopheles* species (2.3% [2/87]) (32 GT); 101 (MT)]. Other *Aedes* species were considered in five inclusions (5.7% [5/87]) [20 (GT); 48 (GM); 79,94,100 (MT)]. There were four inclusions (4.6% [4/87]), three “epidemiological thresholds” [37 (GT)]; 62,65 (MT)] and one “entomological threshold” [96 (MT)] that did not specify any mosquito species.

In parallel with the most reported mosquito species, dengue was the most reported disease (61% [53/87]) [19,22–27,30,33–36,38–44 (GT); 51–60 (GM); 61,64,65,67,69,71,73,75,-77,79,82,84,87–91,93–95,98,99,102 (MT)] followed by chikungunya (10.3% [9/87]) [21,28,31 (GT); 73,81,83,95,99,102 (GM)] and Zika (9.2% [8/87]) [49,50 (GM); 80,81,88,92,95,102 (MT)]. There were eight inclusions for WNV (9.2% [8/87]) (45,46 (GT); 62,63,74,97,103,104 (MT)], three for malaria (3.4% [3/87]) [32,37 (GT); 101 (MT)], two for yellow fever (2.3% [2/87]) 79,91 (MT)], two for Ross river virus (RRV) (2.3% [2/87]) [20 (GT); 100 (MT)], one for Japanese encephalitis (1.1% [1/87]) [29 (GT)], and one for other viral diseases (1.1% [1/87]) [100 (MT)]. Eleven “entomological threshold” inclusions did not specify any disease (12.6% [11/87]) [18,47 (GT); 48 (GM); 66,68,70,72,78,85,86,96 (MT)].

### 5. Variables used in action thresholds/model inputs

Different studies have used different variables to generate thresholds. Mosquito data were the most used (63.2% [55/87]) [21–26,28,30,31,35,36,38,41,43,44 (GT); 55,59 (GM); 61,64,66–89,91–96,98–101,103,104 (MT)] and most of those thresholds were for *Ae*. *aegypti* and/or *Ae*. *albopictus* for the control of dengue (63.6% [35/55]) [22–26,30,35,36,38,41,43,44 (GT); 55,59 (GM); 61,64,67,69,71,73,75–77,79,82,84,87–89,91,93,94,95,98,99 (MT)], chikungunya (14.5% [8/55]) [21,28,31 (GT); 73,81,83,95,99 (MT)], Zika (9% [5/55]) [80,81,88,92,95], yellow fever (3.6% [2/55]) [79,91]. Some of the MTs used mosquito data for thresholds of WNV (5.5% [3/55]) [74,103,104], malaria (1.8% [1/55]) [101], RRV and other diseases (1.8%) [1/55]) [100]. Larval indices (Stegomyia indices- Breteau index, house index, and container index) were the most used mosquito data variable (41.8% [23/55]) [23–26,30,35,36,41,43 (GT); 61,64,71,77,79,82,84,87,88,89,91,92,94,95 (MT)] followed by adult mosquito trap counts (31% [17/55]) [28,44 (GT); 66–68,70,78,80,81,83,86,96,99–101,103,104 (MT)]. The presence of larvae (9% [5/55]) [38 (GT); 59 (GM); 72,74,92 (MT)], pupal index (3.6% [2/55]) [69,75], presence of eggs/egg density (11% [6/55]) [21,31 (GT); 73,76,85,93 (MT)], biting density (1.8% [1/55]) [27 (GT)], resting density (1.8% [1/55]) [24 (GT)], and virus screening (1.8% [1/55]) [55 (GM)] were the other mosquito data variables used. Two other GMs [48,58] used mosquito data only for the development of their climate-based risk prediction models while all other GMs entirely disregarded mosquitoes as either model parameters or output in the development of models.

All the GT “entomological thresholds” and all except Ong et al. [59] of GMs primarily relied on climate variables while none of the MTs reported the use of climate data. Thirty percent of all inclusions (30% [26/87]) [18–20,27,29,32–34,37,39,42,45–47 (GT); 48–58,60 (GM)] used climate variables, mainly temperature (88.5% [23/26]) [18,19,25,27,29,33,39,42,45–47 (GT); 48–58,60 (GM)] and rainfall (53.8% [14/26]) [20,32,34,37,45,46 (GT); 48,52–54,56–58,60 (GM)]. One of those studies [25 (GT)] reported thresholds with several climatic variables; temperature, relative humidity (RH), wind speed, and sunshine duration, and another [57 (GM)] used RH in the final model.

Fifteen of all inclusions (17.2% [15/87]) [40 (GT); 49, 51–57,59,60 (GM); 63,65,90,102 (MT)] used human case data in their thresholds/model inputs with indications of preemptive use of the threshold.

In addition, two inclusions discussed the use of a special index of several variables [63,97 (MT)], and another one used the number of dead birds [62 (MT)]. Geographic location was important in several GMs; Foley and Pecor [50] relied on geo-referencing US military facilities onto published vector and virus habitat suitability maps, while Lowes et al. [52–54] compartmentalized risk to micro-region resolution in Brazil with environmental variables of biome and altitude. One GM that involved spatial environmental parameters in creating risk maps used vegetation, connectivity, and residential coverage [49] while another used terrain height [58]. Zhang et al. [57] stood out because the climate and epidemiological surveillance parameters of one city were modeled to predict the extension of disease transmission risk to a neighboring city. There was a unique GM that approached utilizing social media platform data -Twitter tweets [55].

### 6. Statistical procedures used in GMs

All GMs incorporated a threshold as the dividing value for a binary classification system, i.e., the model output—estimated mosquito count [48], estimated disease cases [51–54,56,57,59,60], probability of epidemic/outbreak [49], probability of exceeding a defined number of disease cases [51–54] that will trigger a warning if the estimate exceeds the binary divider value and thus the area enters a higher category of risk. Some studies involved two or more binary classification schemes that led to multiple risk warning categories (38.5% [5/13]) [50,52–54,59]. Most studies (61.5% [8/13]) directly stated a specific number or rate of disease cases as their chosen EI [51–57,60].

The models vary in complexity and the majority (69.2% [9/13]) involved a generalized linear model or generalized linear mixed model [48,51–58]. A common paradigm was to develop a model that predicted the Y% probability of exceeding the X number of disease cases (i.e., EI). Comparatively, Foley and Pecor [50] created an excel spreadsheet calculator that entailed a monthly review. It ranked Zika transmission risk based on the temperature and habitat suitability of virus and mosquito vectors, meaning four separate binary classifications had to be performed to determine the (forecast) final risk code status for the upcoming month. Other studies utilized Random Forest machine learning [59,60] or developed a risk-assessment framework involving stochastic simulations [49]. A series of inclusions of the same author group detailed the advancing development of a Bayesian spatio-temporal hierarchical model to predict dengue risk in Brazil by microregion [52,53], later tailored to proactively forecast risk for the 2014 World Cup [54], and finally validated after the tournament’s conclusion [64]. The advanced model was also implemented for the island country of Barbados [51]. Interestingly, the advanced model incorporated a pre-defined threshold of cases and a probability threshold that could be optimized to reduce false warnings while aiding policymakers to effectively allocate control resources and activities, a priority also discussed in another GM [49].

### 7. Surveillance and implementation characteristics

Associated surveillance and implementation characteristics reported by GTs/GMs (n = 43) were analyzed. Analysis of MT data were included whenever available. Species-wise analysis was not appropriate due to the very low number of inclusions of other species except *Ae*. *aegypti/Ae*. *albopictus* (80% [36/43]). Hence the analysis was conducted collectively for all inclusions.

#### a. The origin and time span of data

The origin of data used to generate action thresholds varied in different inclusions. All except one inclusion (97.7% [42/43]) used external data sources (previously recorded data) for at least one variable used. Thirty studies (69.8% [30/43]) relied exclusively on external data sources [20, 22–24,27,29,30,32–34,37–40,42–46 (GT); 49–54, 56–60 (GM)] while 13 studies (30.2% [13/43]) [18,21,25,26,28,31,35,36,40,41,47 (GT); 48,55 (GM)] collected self-sampled data. All the self-sampled data were on mosquito data of which 3 collected virus screening data (23% [3/13]) [28,47 (GT); 55 (GM)] and one collected climate data (7.7% [1/13]) [47 (GT)] as well. External data were retrieved on human cases (67.4% [29/43]) [20,23–27,29,30,32,33–39,41,42,44 (GT); 49,51–59 (GM)], climate variables (65% [28/43]) [18–21,24,27–34,37,39,45,46 (GT); 48,50–58,60 (GM)], mosquito data (30.2% [13/43]) [22–24,35,38,41,42,44–46 (GT); 55,58,59 (GM)], and other data such as demographic data, spatial data, tweets (37.2% [10/43]) [49–51,53–55,57–60 (GM)].

More than half of those inclusions (52.2% [23/43]) used >5 years’ data [20,24,27,29–33,35,38–42 (GT); 49,51–54,56,57,59,60 (GM)] with half of them (50% [12/24]) used >10 years’ data [20,29,30,32,38,40 (GT); 49,51,54,56,57,60 (GM)]. Seven studies used 2–5 years’ data (16.3% [7/43]) [34,36,37,46,47 (GT); 48,54 (GM)] and 12 studies (28% [12/43] used data spanning less than two years [18,19,21–23,26,28,31,43,44 (GT); 50,58 (GM)]. The majority of MTs originated before 2010 (52.3% [23/44]) [61–65,67–69,71,75,76,79,81,82,84,87,88,89,91–93,95,100,104] while the time of origination was not clear in other MTs.

#### b. Data frequency

The majority of GTs and GMs that used mosquito data relied mostly on daily/weekly data (35.3% [6/17]) [21,23,28,31,44 (GT); 55 (GM)] or bi-weekly/monthly data (35.3% [6/17]) [24,25,30,35,36,41 (GT)]. Two inclusions used yearly data (11.8% [2/17]) [38 (GT); 59 (GM)], one inclusion used only one-time data to generate action thresholds for two city parks (6% [1/17]) [22 (GT)], and there were two inclusions with no clear mention of the data frequency (11.8% [2/17]) [26,43 (GT)]. Inclusions that used climatic variables relied mostly on daily/weekly data (61.5% [16/26]) [18,19,27,33,34,42,45–47 (GT); 48,49,55–58,60 (GM)] over monthly data (38.5% [10/26]) [20,29,32,37,39 (GT); 50–54 (GM)]. Daily/weekly data frequency was the most used (50% [5/10]) [49,55–57,60 (GM)] in GTs and GMs that used human case data.

#### c. Spatial unit of analysis and intended spatial implementation of thresholds

The spatial assessment level (data collection units) of GTs/GMs varied from 1x1 km grid cells in neighborhoods to district blocks, to represent the same or larger spatial units of analysis (study area). Town/city (28% [12/43]) [21,24,27,30,33,40,44 (GT);55–57,59,60 (GM)], country (14% [6/43]) [29,32,42 (GT); 51,52,54 (GM)], municipality (9.3% [4/43]) [18,25,26,47 (GT)], district (9.3% [4/43]) [23,36,37,43 (GT)], and region (9.3% [4/43]) [34,45,46 (GT): 53 (GM)] were the most studied spatial units of analysis.

Those thresholds were generated with the intention of implementing them in the study area itself (69.7% [30/43]) [22,23,25–27,29,31–34,36–46 (GT); 49–51,54,56–60 (GM)] or a larger area including the study area (28% [12/43]) [18,19,21,24,28,30,35,47 (GT); 48,52,53,55 (GM)]. Only one inclusion intended to implement the threshold in a smaller area than the study area (2.3% [1/43]) [20 (GT)].

More than half of the MTs (66% [29/44]) [61–65,66–68,70,72–74,76–78,80,83,85–87,89,90,93,96,97,100,101,103,104] mentioned thresholds that originated in the same country or at least in the same region [102] and of these, nine originated from the same individual state within the United States [63,68,72,74,78,86,96,97,103]. Eight inclusions (18.2% [8/44]) [66,78,86,90,96,100–102] mentioned thresholds that originated from the same program. One inclusion (2.3% [1/44]) [75] mentioned a threshold with a different country origin while four inclusions (9% [4/44]) [69,88,92,94] mentioned thresholds from different continental regions. Only one inclusion (2.3% [1/44]) [81] mentioned different thresholds either with the same country origin or different country origin and one inclusion (2.3% [1/44]) [98] did not cite a cross-reference or mention the origin of the mentioned threshold. Globally recognized thresholds were mentioned in six inclusions (11.4% [5/44]) [71,79, 84,91,95].

#### d. Lead time and validation of the threshold

The most reported lead time that used mosquito data (41.2% [7/17]) [24,30,35,36,41,44 (GT); 55 (GM)], as well as climate data (46.2% [12/26]) [20,27,33,37,39,42,45 (GT); 51,54,55,57,60 (GM)], was 1–3 months. Three inclusions with mosquito data (11.8% [2/17]) [24 (GT); 55 (GM)] and three inclusions with climate data (11.5% [3/26]) [18 (GT); 55,58 (GM)] reported 1–3 weeks lead time. Using climate data, four other inclusions (15.4% [4/26]) [52,53,56,57 (GM)] reported a lead time of more than 3 months while Smith et al. [32 (GT)] reported a different lead time. However, ten mosquito data inclusions (58.8% [10/17]) [21–23,25,26,28,31,38,43 (GT); 59 (GM)] did not report any lead time. Notably, several GMs utilized or made suggestions to utilize a specific climate forecasting resource to optimize the proactive lead time for control interventions [50–54]. Others developed models that used climate data at time points prior to the time point of model predictions, although lead-time would only be as long as the shortest time-lagged variable [49,56,57].

Most inclusions (60.5% [26/43]) analyzed performance statistics for GT/GM validation purposes. Of those, the majority used only training data (57.7% [15/26]) [20,23–25,27,31,32,38,40,41,45 (GT); 51,52,54,57 (GM)] while only eleven inclusions (42.3% [11/26]) [21,36,43,46 (GT); 48,53,55,56,58–60 (GM)] used testing data worth 6 weeks to 5 years. Of all GTs/GMs seventeen (39.5% [17/43]) [18,19,22,26,28–30,33–35,37,39,42,44,47 (GT); 49,50 (GM)] did not report on validation of GT/GM performance. However, some of those GTs were validated in subsequent studies which were included in the review as MTs [61–65].

### 8. Intended use of the threshold

Two broad categories of intended uses were identified from the inclusions. Direct intentions included those that stated direct utility for initiating mosquito control operations by setting an operational target threshold to keep populations below unaccepted levels of biting nuisance/disease transmission and others that determined different risk levels to guide a mosquito control response and/or effective resource allocation. Indirect intentions involved inclusions that mentioned mosquito/vector control at least once but discussed the utility of the action threshold within the wider expanse of a public health response without a specific discussion of focused implementation in mosquito control.

The majority of inclusions (88.5% [77/87]) [18–23,25–28,31,34–36,38,40–47 (GT); 48,50,52–60 (GM); 61–64, 66–104 (MT)] indicated the direct use of the threshold in mosquito control. Seven inclusions (11.5% [10/87]) 24,29,30,32,33,37,39 (GT); 49,51 (GM); 65 (MT)] intended to use the thresholds in initiating or guiding public health responses to control disease spread indirectly indicating their use in mosquito control.

## Discussion

Although the establishment of action thresholds has become critically important to achieve the maximum cost-effective benefit of mosquito control interventions, there is no formulated guidelines on the development of thresholds in different environmental settings. This review attempted to identify publications with mosquito control action thresholds across the world during the last 10 years and the basic surveillance and implementation characteristics associated with those thresholds. We hope the findings of the review will help guide mosquito control program managers and any other personnel who are interested in developing/using action thresholds to be included in their programs.

The 87 inclusions of the review were categorized into three broad categories; originally generated thresholds, generated models, and mention of previously generated thresholds. General information and surveillance/implementation characteristics of the three categories were examined to understand the structure of their surveillance systems.

More than 90% of the inclusions were from scientific journals/conference proceedings indicating the scientific/academic interest on developing action thresholds. However, the very low number (6.8%) of inclusions from program guidelines/technical reports could not be considered due to the scarcity of thresholds being used practically in mosquito control programs. There could be established thresholds that have already been in the specific IMM toolboxes of mosquito control programs but not disseminated in publications or in publications that fall into our inclusion criteria. We have planned and already initiated to identify those action thresholds through individual discussions with program managers/authorities in another study.

Overall, 77% of identified thresholds were “epidemiological thresholds” and thus, indicate that the need to establish mosquito control action thresholds has been driven mainly by the transmission dynamics of mosquito-borne diseases rather than by biting nuisance. The majority of epidemiological thresholds here were to indicate transmission of disease although there is a chance of overlapping as the used epidemiological indication terms were not based on strict definitions. It is evidence for the need of more promising proactive mosquito control as discussed by Eisen et al. [105] rather than waiting for the transmission to transform into outbreaks or epidemics. Although mosquitoes are widely distributed in different climatic regions in the world, they thrive in regions with warm temperatures, humid conditions, and high rainfall, and thus tropical and sub-tropical areas are ideal for their survival [106]. Most of the identified action thresholds (>70%) being generated in tropical and sub-tropical climatic regions indicate their relevance to the burden of mosquitoes and mosquito-borne diseases in those regions, compared to others. The fact that the highest number of thresholds, were originated in Asia and associated most with dengue and dengue vectors should have a link with recent increases in the incidences of major dengue outbreaks and epidemics reported mainly in the Asian region [107,108]. However, recent studies show the geographical distribution of *Ae*. *aegypti* and *Ae*. *albopictus* is now extensive in all human inhabited continents; Europe and North America [109], South America [110], Oceania [111,112], and Africa [113].

Overall, the majority of inclusions have used mosquito data in thresholds. However, some of the recently developed entomological thresholds have moved on to use climate data, mainly temperature, rather than using mosquito data itself as done previously (before 2010). It is a good step forward to proactively inform the need to initiate mosquito control and thus help bring about more effective prevention of unacceptable levels of mosquito abundance. Despite controversy on the association with the number of disease cases [36,41,114–122], the majority of epidemiological thresholds for dengue and chikungunya were generated using Stegomyia larval indices.

The data collection duration is a substantial characteristic in the process of action threshold development. A sufficient time span would allow identifying any time sensitive variations in the identified correlations, thus in the generated action thresholds. Therefore, thresholds developed using historic data would be more reliable than those generated using only current data or one-time data. Davis et al. [123] suggested the inconsistent reliability and questionable validity of the thresholds generated in the study by using <3 years data would be refined with additional years of data. Using <5 years’ historic data in their study, Bowman et al. [124] suggested that data of a greater number of historic years would have generated more reliable results. Other studies which reported alert thresholds for malaria based on disease case numbers discuss the need of at least 5 years of historical data [125,126]. The majority of studies reviewed here being used carried out over more than 5 years have allowed the possible temporal variations to be considered and compromised. Studies conducted in less than 2 years might not be able to catch time sensitive variations in the variables used thus making the threshold less reliable to use over the years. Likewise, mosquito abundance and disease transmission dynamics may change over time due to many reasons such as environmental/climatic changes, human mobility, etc. Most of the “mentioned thresholds” being developed more than ten years ago might need assessments and necessary updates before using in current mosquito control programs. Notably, almost all identified studies have used external data sources mainly from relevant government entities to generate thresholds. The use of external data sources requires careful selection and matching of data in the temporal and spatial units in concern to generate reliable thresholds. The distribution of mosquitoes and hence the mosquito-borne diseases are clustered in nature based on different ecological niches they use [127]. However, the demarcation of spatial units of those studies is based on man-made administrative boundaries rather than on ecological or geographical determinants. Therefore, the use of a particular action threshold in the spatial unit of its origin is more reasonable than using it in an area with different geographical, climatic, environmental, demographic, socio-economic, and other relevant characteristics. Stegomyia house index (HI) of less than 1% indicates no risk of a dengue outbreak in Brazil [128] but outbreaks occurred in Singapore when the national overall HI was less than 1% [129]. Researchers have shown that the geographical level heterogeneity affects the performance of thresholds with the thresholds developed for smaller units performing best [120]. The thresholds varied among different spatial units such as different districts in the same country [37,41] as well as among different climatic zones in the same country [39]. Romiti et al. [130] report a significant difference in *Ae*. *albopictus* development threshold temperature among municipalities. In contrast, some studies report the same threshold for the seasonal emergence of *Ae*. *albopictus* in two countries [18,47]. Bowman et al. [124] report the predictive ability of certain meteorological and epidemiological alarm variables of dengue across all countries studied. Hence, there could not be universally reliable thresholds and there is no definitive fact that the same threshold cannot be used elsewhere. The majority of the publications analyzed here have action thresholds intended to be used in the same spatial unit as its origin. However, locations with similar determinant characteristics but yet unable to collect required surveillance data would need to consider using an already developed appropriate threshold after confirming with initial environmental assessments/validations.

Temporal resolution of data collection and analysis plays an important role in the development of reliable action thresholds. Smaller scale resolutions (e.g. weekly data) would reveal more precise relationships between variables compared to higher scale resolutions (e.g. monthly/yearly data) [131]. Realistically, the available resources and other logistics will be deterministic factors of the temporal resolution of data collection. However, most of the studies included in this review achieved at least a monthly resolution.

One of the main purposes of establishing mosquito control action thresholds is to provide sufficient time for planning, organizing, and implementing appropriate control activities at the optimal time. Sufficient lead time from the established threshold to the exceedance of its indication would help mosquito control program managers to plan, organize and implement appropriate control measures for effective reduction of mosquito populations. Entomological thresholds seem to have shorter lead time (mentioned in only 2 studies) than epidemiological thresholds. Epidemiological thresholds which used climatic variables have longer lead times than those using mosquito data variables. Hii et al. [132] discuss the importance of the optimal lead time which would give sufficient time for successful implementation of mosquito control. They suggest that an average of 2 months with maximum 3 months lead time is typically required for effective mitigation of dengue transmission in Singapore in both non-epidemic and epidemic years. The development of a reliable mosquito control action threshold/s is a very complex process due to the interplay of several tangential factors, such as frequency and amount of virus importation, and herd immunity [133] and human-mosquito contact rates [134]. However, those thresholds should be accurate enough not to provide too many false warnings which would lead to issues such as wastage of resources and development of insecticide resistance. Validation of generated thresholds is important before establishing to confirm their performance in operational settings. Validation studies have confirmed the effectiveness of thresholds generated in the same study [36] as well as previous thresholds [49]. After field validation of previous thresholds by Sanchez et al. [120], the performance of overall neighborhood Breteau Index (BI) ≥ 1.3 was denied and the most straight forward performance of BI_max_ ≥ 4 was confirmed as an indicator for predicting dengue transmission [48]. MacCormack-Gelles et al. [51] showed that the HI threshold imposed by the Brazilian Ministry of Health was not in compliance with increasing dengue risk in Fortaleza, Brazil. Most of the thresholds included in this review still need to be field validated, before establishing to inform the need of initiation of mosquito control activities.

### Limitations

Pertaining to the restrictions of available time and resources, the systematic review has several limitations which might have eliminated some important publications: (i) the literature search was limited only to two search engines (Google scholar and PubMed), (ii) the search was limited to 2010–2021, (iii) language restricted to English only, and (iv) grey literature not used.

### Conclusions

- Identification of (i) different action thresholds in existence for different mosquito species and different related diseases in different countries (S1,S2 and S3 Tables), and (ii) the characteristics associated in generating them will help interested mosquito control program managers and others to better plan their surveillance systems for required data collection.
- The results give an insight to the possibility of moving on to climatic variables rather than mosquito data variables itself to generate action thresholds which would give longer lag time for the required preparations to implement control activities.
- The review uncovers (i) the gap in the literature on action thresholds for mosquito nuisance, other mosquito-borne- diseases than dengue in Asia, mosquito-borne diseases in other geographic regions and (ii) the need to update previous action thresholds in space and time.

## Supporting information

S1 Checklist PRISMA checklist for abstracts. (DOCX)

S2 Checklist PRISMA checklist. (DOCX)

S1 Table Generated mosquito control action thresholds (“generated thresholds”) found in publications included in the review. (XLSX)

S2 Table Generated models for mosquito control action thresholds (“generated-models”) found in publications included in the review. (XLSX)

S3 Table Mentioned mosquito control action thresholds (“mentioned thresholds”) found in publications included in the review. (XLSX)
